# Screening for Hepatic Steatosis and Fibrosis Among Physicians Attending the Integrated Diabetes and Endocrine Academy Conference: A Cross-Sectional Study

**DOI:** 10.7759/cureus.110948

**Published:** 2026-06-16

**Authors:** Binayak Sinha, Samit Ghosal, Soumyabrata RoyChaudhuri, Anirban Majumder, Ajoy Biswas, Awadhesh K Singh, Biswajit Ghosh Dastidar, Debasish Maji, Ghanshyam Goyal, JJ Mukherjee, Kalyan K Gangopadhyay, Masood Batin, Sanjay Chatterjee, Subir Ray, Sudip Chatterjee, Sujoy Majumdar

**Affiliations:** 1 Endocrinology, Manipal Hospital, Kolkata, IND; 2 Endocrinology, Nightingale Hospital, Kolkata, IND; 3 Endocrinology, Kali Prasad Chowdhury Medical College & Hospital, Kolkata, IND; 4 Internal Medicine, GD Hospital and Diabetes Institute, Kolkata, IND; 5 Diabetes and Endocrinology, GD Hospital and Diabetes Institute, Kolkata, IND; 6 Internal Medicine, Woodlands Multispeciality Hospital, Kolkata, IND; 7 Endocrinology, Vivekananda Institute of Medical Sciences, Kolkata, IND; 8 Endocrinology, ILS Hospital, Kolkata, IND; 9 Endocrinology, Apollo Multispeciality Hospital, Kolkata, IND; 10 Endocrinology, Peerless Hospital, Kolkata, IND; 11 Cardiology and Diabetology, Mercy Hospital, Kolkata, IND; 12 Diabetology, Apollo Multispeciality Hospital, Kolkata, IND; 13 Endocrinology and Diabetes, Apollo Multispeciality Hospital, Kolkata, IND

**Keywords:** fibrosis-4 score, ideacon, metabolic dysfunction-associated steatotic liver disease (masld), physicians, transient elastography (fibroscan)

## Abstract

Introduction

Metabolic dysfunction-associated steatotic liver disease (MASLD) is commonly detected using the Fibrosis-4 (FIB-4) score, a non-invasive tool for advanced fibrosis, though its screening utility in this specific population remains uncertain. Transient elastography (FibroScan) measures steatosis using the controlled attenuation parameter (CAP) and fibrosis using liver stiffness measurement (LSM), both of which remain underexplored in metabolic diseases. This study evaluated the effectiveness of FIB-4 in screening for advanced hepatic fibrosis 3 (F3) and advanced hepatic fibrosis 4 (F4) in physicians with generalised or central obesity.

Methods

Physicians (n = 162) at the Integrated Diabetes and Endocrine Academy Conference (IDEACON) were assessed for demographics (age, sex), anthropometrics (height, body mass index (BMI), waist circumference (WC)), laboratory parameters (alanine aminotransferase (ALT), aspartate aminotransferase (AST), glycated haemoglobin (HbA1c), platelet count), and FIB-4. CAP and LSM were measured using FibroScan, and the diagnostic performance of FIB-4 was evaluated against CAP and LSM-defined outcomes for hepatic steatosis and fibrosis.

Results

The mean age of the participants was 46.0 years, and 86.3% were male. The mean BMI was 27.6 kg/m², and the prevalence of type 2 diabetes mellitus (T2DM) was 28.6%. FIB-4 showed poor sensitivity (6.3% for LSM F3/F4 and 5.1% for stage 3 (S3) fibrosis) and area under the curves (AUCs) (0.51, 0.49), despite high specificity (95.7%). Multivariate analysis identified WC (OR = 1.09, p = 0.036) and ALT (OR = 1.05, p = 0.013) as predictors of CAP S3, age (OR = 1.16, p = 0.021) for LSM F3, and T2DM (OR = 33.34, p = 0.010) for LSM F4. Obese participants (88.8%) had higher CAP S3 (25.7%) and LSM F3/F4 (18.1%).

Conclusion

FIB-4 is inadequate for screening hepatic fibrosis in physicians. Combined CAP and LSM assessment is essential for at-risk individuals with obesity and increased WC.

## Introduction

Metabolic dysfunction-associated steatotic liver disease (MASLD), a global health concern with ~30% prevalence driven by obesity, spans simple steatosis to non-alcoholic steatohepatitis (NASH), advanced fibrosis, cirrhosis, and hepatocellular carcinoma [1,2]. Liver biopsy, though the gold standard for early detection, is invasive, costly, and impractical for screening [3]. Non-invasive Fibrosis-4 (FIB-4) score, which uses age, alanine aminotransferase (ALT), aspartate aminotransferase (AST), and platelet count, is gaining traction for identifying advanced fibrosis due to its simplicity [4]. Yet its utility in high-risk obese or metabolic groups is uncertain [5], with ~7.8% false negatives (FIB-4 <1.3) in advanced fibrosis [6].

Transient elastography (FibroScan) measures liver stiffness for fibrosis using liver stiffness measurement (LSM) and controlled attenuation parameter (CAP) for steatosis, with validated cutoffs, i.e., CAP >280 dB/m for stage 3 (S3) and LSM >12.5 kPa for advanced hepatic fibrosis 4 (F4) [7,8]. Though effective, its limited accessibility relative to blood-based FIB-4 fuels debates over screening strategies [9].

Physicians themselves face these risks, yet few studies assess their hepatic health [10].

The primary objective of this study was to evaluate the diagnostic performance of the FIB-4 score for detecting advanced hepatic fibrosis (F3/F4) using transient elastography among physicians attending the Integrated Diabetes and Endocrine Academy Conference (IDEACON).

The secondary objectives were (1) to determine the prevalence of hepatic steatosis and fibrosis using CAP and LSM measurements; (2) to identify clinical, anthropometric, and biochemical predictors associated with steatosis and fibrosis; (3) to explore the potential value of combined CAP and LSM assessment in physicians with metabolic risk factors, including obesity and increased waist circumference (WC).

## Materials and methods

Study design and population

The study was conducted during an annual national conference on diabetes and endocrinology organised by the Integrated Diabetes and Endocrine Academy (IDEA) in July 2024 at the conference venue in Kolkata, where interested physicians were enrolled in the study. The study included 162 adult physicians who voluntarily participated in a health screening programme conducted during IDEACON 2024. Participation was open to all attending physicians. Although the screening initiative was primarily promoted among individuals with metabolic risk factors, including obesity and increased WC, enrolment was not restricted to these groups. Therefore, both obese and non-obese physicians were included in the final analysis. This cross-sectional analytical study was conducted to evaluate the utility of the FIB-4 score in detecting hepatic fibrosis and to determine the necessity of dual screening with CAP and LSM in at-risk individuals.

Inclusion criteria included the following: (1) adult physicians attending IDEACON 2024 who voluntarily agreed to participate; (2) availability of anthropometric measurements, laboratory investigations, and FibroScan assessment; (3) documented negative viral screening for HBV or HCV within the preceding six months.

Exclusion criteria included the following: (1) positive viral screening for HBV or HCV; (2) alcohol consumption more than 70 g/week for women, and more than 140 g/week for men.

Physicians attending IDEACON 2024 were invited to participate voluntarily in this study. All participants underwent blood sampling and transient elastography after a minimum fasting period of three hours. The same fasting protocol was applied uniformly to all participants to minimise postprandial variability in liver stiffness and CAP measurements while ensuring feasibility within the conference-based screening programme. FibroScan was performed using the Echosens FibroScan Mini+ 430 machine (Echosens, Paris, France). M+ probe (3.5 MHz) was used in non-obese adults with skin to capsule distance less than 2.5 cm, whereas XL+ probe (2.5 MHz) was used in obese adults where skin to capsule distance was greater than 2.5 cm. All transient elastography examinations were performed by a single trained operator. A minimum of 10 valid measurements was required for each examination, and only studies with an interquartile range-to-median ratio (IQR/M) <30% were considered acceptable, in accordance with standard quality criteria. Blood samples were collected and analysed in a single National Accreditation Board for Testing and Calibration Laboratories (NABL)-accredited laboratory, using the Cobas 6000 analyser (Roche Diagnostics, Rotkreuz, Switzerland) for liver parameters, and platelet count was tested using SYSMEX XN-550 cell counter (Kobe, Japan). All data were collected manually and, after depersonalisation, were formatted in an Excel sheet (Microsoft Corporation, Redmond, WA) and sent for statistical evaluation. The CAP and LSM cut-offs were taken as per the European Association for the Study of the Liver (EASL) clinical practice guidelines [8]. As this study was conducted as a conference-based cross-sectional screening initiative, no formal sample size calculation was performed a priori. All eligible physicians who voluntarily consented to participate during the study period were included in the analysis.

Data collection and measurements

Demographic data (age, sex), anthropometric measurements (height, weight, WC), systolic blood pressure (SBP), diastolic blood pressure (DBP), and laboratory parameters (ALT, AST, platelet count, glycated haemoglobin (HbA1c)) were collected. Height and weight were measured, and BMI was calculated as weight (kg) divided by height squared (m²). WC was measured at the midpoint between the lower rib and iliac crest with a non-stretchable measuring tape. Type 2 diabetes mellitus (T2DM) status was recorded as a binary variable (yes/no) based on a previous diagnosis of diabetes mellitus and/or an HbA1c value consistent with diabetes (HbA1c ≥6.5%) according to accepted diagnostic criteria. Previously diagnosed and newly identified cases were analysed together as a single T2DM category. Information regarding the duration of diabetes or whether diabetes was newly detected during screening was not systematically collected, and therefore separate analyses were not performed. Only physicians with documented negative viral screening for HBV and HCV within the previous six months were included. Liver health was evaluated using FibroScan by a single operator who provided both CAP and LSM values. CAP was employed to quantify hepatic steatosis and was categorised as stage 0 (S0; <248 dB/m), stage 1 (S1; 248-268 dB/m), stage 2 (S2; 269-280 dB/m), and stage 3 (S3; >280 dB/m). LSM was utilised to stage hepatic fibrosis as no fibrosis-mild fibrosis (F0-F1; <7.0 kPa), moderate fibrosis (F2; 7.0-9.5 kPa), advanced hepatic fibrosis (F3; 9.6-12.5 kPa), and advanced hepatic fibrosis (F4; >12.5 kPa). The FIB-4 score was calculated using age, ALT, AST, and platelet count [4].

Statistical analysis

All statistical analyses were conducted using DATATab software (numiqo e.U., Graz, Austria). Descriptive statistics summarised the data, with continuous variables expressed as means, standard deviation (SD), or medians; IQR was based on the normality assessment performed with the Kolmogorov-Smirnov test. Categorical variables were presented as percentages.

To evaluate the diagnostic performance of the FIB-4 score for detecting advanced hepatic fibrosis, sensitivity, specificity, positive predictive value (PPV), negative predictive value (NPV), false positive rate (FPR), and false negative rate (FNR) were calculated using transient elastography-defined fibrosis stages as the reference standard. Receiver operating characteristic (ROC) curve analysis was performed, and the area under the curve (AUC) was determined to assess the discriminatory ability of FIB-4 for advanced fibrosis and steatosis.

For association analyses, univariate and multivariate linear regression models were used when hepatic outcomes were analysed as continuous variables, specifically CAP score (steatosis burden) and LSM score (fibrosis burden). Candidate variables for multivariable analyses were selected based on their established clinical relevance to MASLD and fibrosis risk, as well as their availability in the study dataset. Unstandardised beta coefficients (B), standard errors (SE), and p-values were reported for these models. Unstandardized coefficients were preferentially reported to enhance clinical interpretability and maintain consistency with standard errors and confidence intervals across linear regression models. Binary logistic regression analyses were separately performed for categorical hepatic outcomes, including CAP S3 and LSM F3/F4, to identify independent predictors; these results were reported as odds ratios (ORs) with 95% confidence intervals (CIs). Pearson correlation coefficients were calculated to assess relationships between continuous anthropometric and hepatic variables. Statistical significance was defined as a two-tailed p-value < 0.05.

## Results

Baseline characteristics of the participants

The cohort had a mean age of 46.02 years (SD = 15.87) and was predominantly male (86.34%, n = 140; 95% CI = 80.2-91.1). The mean BMI was 27.61 kg/m² (SD = 4.05), with 88.82% (n = 144; 95% CI = 82.8-93.2) classified as obese (BMI ≥23 kg/m²). WC averaged 95.76 cm (SD = 11.19) in males and 91.5 cm (SD = 11.94) in females. T2DM was present in 28.57% (n = 46) (Table 1).

**Table 1:** Baseline characteristics of 162 participants. BMI = body mass index; WC = waist circumference; SBP = systolic blood pressure; DBP = diastolic blood pressure; T2DM = type 2 diabetes mellitus; HbA1c = glycated haemoglobin; TG = triglycerides; ALT = alanine aminotransferase; AST = aspartate aminotransferase; FIB-4 = Fibrosis 4; CAP = controlled attenuation parameter; S0 = stage 0; S1 = stage 1; S2 = stage 2; S3 = stage 3; LSM = liver stiffness measurement; F0-F1 = no fibrosis-mild fibrosis; F2 = moderate fibrosis; F3 = advanced hepatic fibrosis; F4 = advanced hepatic fibrosis.

Variable	Mean (SD)	Median (IQR)	Percentage
Age (years)	46.02 (15.87)		
Sex (male)			86.34%
Height (cm)	164.71 (7.58)		
Weight (kg)	75.15 (13.49)		
BMI (kg/m^2^)	27.61 (4.05)		
BMI <23 kg/m^2^			11.18%
WC (cm): male	95.76 (11.19)		
WC (cm): female	91.5 (11.94)		
SBP (mm of Hg)		128.87 (20)	
DBP (mm of Hg)		80 (5)	
T2DM (yes)			28.57%
HBA1c (%)		5.5 (0.9)	
TG (mg/dL)	205.27 (103.73)		
Creatinine (mg/dL)		0.92 (0.2)	
ALT (IU/L)		28 (22)	
AST (IU/L)		24 (11)	
Platelet count (x10^9^/L)		211 (79)	
FIB-4 score		0.9 (1)	
FIB-4 <1.3			63.98%
FIB-4 1.3-2.67			32.92%
FIB-4 >2.67			3.11%
CAP score	253.99 (49.66)		
CAP S0			38.51%
CAP S1			20.5%
CAP S2			16.77%
CAP S3			24.22%
LSM score		6.1 (2.8)	
LSM F0-F1			69.57%
LSM F2			13.66%
LSM F3			7.45%
LSM F4			9.32%

Screening utility of the FIB-4 score

**Figure 1:**
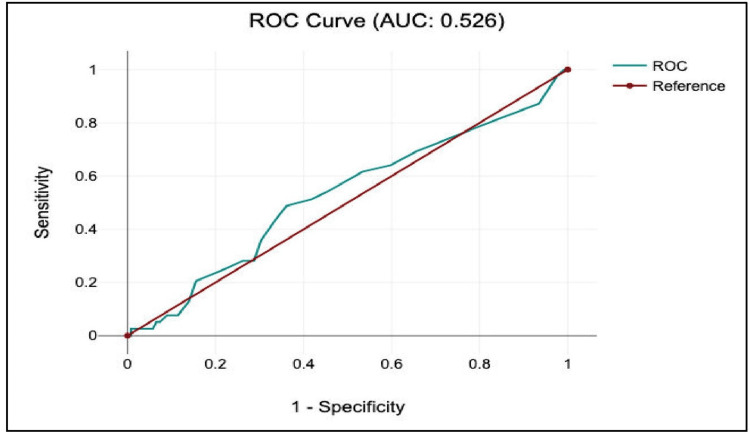
Receiver operating characteristic (ROC) curve of the FIB-4 score for detecting advanced fibrosis (LSM F3/F4). Area under the curve (AUC) = 0.51 (95% CI = 0.40-0.62). LSM = liver stiffness measurement.

The FIB-4 score’s performance in detecting fibrosis (LSM F3/F4) was assessed using LSM as the reference standard for fibrosis (Table 2). In 64% of the subjects, the FIB-4 score was < 1.3. FIB-4 score was indicative of fibrosis in 32.9% of the participants and suggestive of advanced fibrosis in 3.1%. FibroScan was indicative of advanced fibrosis (F3/F4) among 16.8% of subjects (24 out of 162). For advanced fibrosis (F3/F4, n = 27; 16.77%, 95% CI = 11.3-23.5), FIB-4 (>2.67 vs. <1.3) exhibited low sensitivity (6.3%, 95% CI = 0.8-20.8) and high specificity (95.7%, 95% CI = 90.8-98.5), with a PPV of 20.0% (95% CI = 2.5-55.6) and NPV of 85.4% (95% CI = 79.0-90.5). The FNR was 93.8% (95% CI = 79.2-99.2), indicating poor detection of actual cases. The AUC was 0.51 (95% CI = 0.40-0.62), suggesting minimal discriminatory power (Figure 1). For advanced steatosis (CAP S3, n = 39; 24.22%, 95% CI = 17.8-31.6), FIB-4 showed similarly poor sensitivity (5.1%, 95% CI = 0.6-17.3) and an AUC of 0.49 (95% CI = 0.39-0.59), reinforcing its futility as a screening tool for either condition.

**Table 2:** Screening performance of the Fibrosis-4 (FIB-4) score for hepatic outcomes in physicians attending IDEACON (n = 162). Metrics are presented as percentages with 95% CIs for the FIB-4 score (cutoffs: <1.3, negative; >2.67, positive) in detecting advanced fibrosis (LSM F3/F4) and steatosis (CAP S3). TPR = true positive rate (sensitivity); TNR = true negative rate (specificity); FPR = false positive rate; FNR = false negative rate; PPV = positive predictive value; NPV = negative predictive value; LSM = liver stiffness measurement; CAP = controlled attenuation parameter; IDEACON = Integrated Diabetes and Endocrine Academy Conference.

Screening metric	% (95% CI)	Interpretation
TPR (sensitivity)	6.3 (0.8-20.8)	Low ability to detect advanced fibrosis
TNR (specificity)	95.7 (90.8-98.5)	High ability to rule out fibrosis absence
FPR	4.3 (1.5-9.2)	Low rate of incorrect fibrosis detection
FNR	93.8 (79.2-99.2)	High rate of missed fibrosis cases
PPV	20.0 (2.5-55.6)	A low probability of a positive indicates fibrosis
NPV	85.4 (79.0-90.5)	A high probability of a negative excludes fibrosis

Associations with hepatic steatosis and fibrosis

Univariate Linear Regression Analysis for Continuous CAP and LSM Outcomes

Univariate linear regression analyses were performed to assess associations between clinical/anthropometric variables and CAP and LSM score outcomes among 162 physicians attending IDEACON (Table 3). B, SE, and p-values are reported for each predictor.

**Table 3:** Univariate linear regression associations between clinical variables and continuous CAP and LSM scores in physicians attending IDEACON (n = 162). Univariate linear regression results demonstrate associations between clinical/anthropometric predictors and continuous CAP and LSM scores. Results are presented as unstandardised regression coefficients (B), standard errors (SE), and p-values. CAP = controlled attenuation parameter; LSM = liver stiffness measurement; WC = waist circumference; BMI = body mass index; SBP = systolic blood pressure; DBP = diastolic blood pressure; ALT = alanine aminotransferase; AST = aspartate aminotransferase; T2DM = type 2 diabetes mellitus; TG = triglycerides; HbA1c = glycated haemoglobin; IDEACON = Integrated Diabetes and Endocrine Academy Conference.

Variable	CAP score (Linear regression: B, SE, p)	LSM score (Linear regression: B, SE, p)
	B (SE, p-value)	B (SE, p-value)
Age (years)	0.40 (0.25, p=0.103)	0.00 (0.02, p=0.998)
Sex (male)	19.42 (11.33, p=0.088)	1.67 (1.01, p=0.098)
Height (cm)	1.30 (0.51, p=0.012)	0.04 (0.05, p=0.427)
Weight (kg)	1.15 (0.28, p<0.001)	0.08 (0.03, p=0.001)
WC (cm)	1.42 (0.33, p<0.001)	0.06 (0.03, p=0.046)
BMI (kg/m²)	3.18 (0.94, p=0.001)	0.29 (0.08, p=0.001)
BMI ≥23 kg/m²	34.39 (12.16, p=0.005)	1.48 (1.10, p=0.179)
SBP (mm of Hg)	0.40 (0.33, p=0.24)	0.04 (0.03, p=0.228)
DBP (mm of Hg)	0.88 (0.61, p=0.149)	0.04 (0.05, p=0.495)
ALT (U/L)	0.60 (0.15, p=0.001)	0.03 (0.01, p=0.014)
AST (U/L)	0.99 (0.30, p=0.001)	0.06 (0.03, p=0.025)
Platelet count (*10^9^/l)	0.02 (0.05, p=0.705)	0.01 (0.00, p=0.207)
T2DM (Yes)	17.45 (8.58, p=0.044)	1.50 (0.76, p=0.051)
TG (mg/dl)	0.08 (0.04, p=0.038)	0.00 (0.00, p=0.527)
HbA1c (%)	8.58 (4.05, p=0.036)	0.78 (0.36, p=0.032)
Creatinine (mg/dl)	4.29 (10.96, p=0.696)	-0.44 (0.97, p=0.652)

For CAP score, significant positive associations were observed with height (B = 1.30, SE = 0.51, p = 0.012), weight (B = 1.15, SE = 0.28, p < 0.001), WC (B = 1.42, SE = 0.33, p < 0.001), BMI (B = 3.18, SE = 0.94, p = 0.001), and BMI ≥23 kg/m² (B = 34.39, SE = 12.16, p = 0.005). These findings indicate that each 1-cm increase in height, each 1-kg increase in weight, each 1-cm increase in WC, and each 1-kg/m² increase in BMI were associated with corresponding increases of 1.30, 1.15, 1.42, and 3.18 CAP units, respectively, while participants with BMI ≥23 kg/m² had CAP scores 34.39 units higher than those with BMI <23 kg/m². Additional significant predictors included ALT (B = 0.60, SE = 0.15, p = 0.001), AST (B = 0.99, SE = 0.30, p =0.001), T2DM (B = 17.45, SE = 8.58, p = 0.044), and HbA1c (B = 8.58, SE = 4.05, p = 0.036). Sex (Male) showed a borderline association (B = 19.42, SE = 11.33, p = 0.088), while age and platelet count were not significant (p > 0.05).

For LSM score, significant associations were found with weight (B = 0.08, SE = 0.03, p = 0.001), WC (B = 0.06, SE = 0.03, p = 0.046), BMI (B = 0.29, SE = 0.08, p = 0.001), ALT (B = 0.03, SE = 0.01, p = 0.014), AST (B = 0.06, SE = 0.03, p = 0.025), and HbA1c (B = 0.78, SE = 0.36, p = 0.032). These findings indicate that each 1-kg increase in weight, each 1-cm increase in WC, each 1-kg/m² increase in BMI, each 1-U/L increase in ALT, each 1-U/L increase in AST, and each 1% increase in HbA1c were associated with corresponding increases of 0.08, 0.06, 0.29, 0.03, 0.06, and 0.78 units in LSM score, respectively. T2DM was borderline (B = 1.50, SE = 0.76, p = 0.051), and sex (male) approached significance (B = 1.67, SE = 1.01, p = 0.098). No significant associations were observed with age, height, BMI ≥23 kg/m², or platelet count (p > 0.05).

Multivariate logistic regression analysis

Multivariate regression analyses were conducted to assess the predictors of continuous CAP S3 and LSM F3/F4 among 162 physicians attending IDEACON, adjusting for age, sex, height, weight, WC, BMI, SBP, DBP, ALT, AST, platelet count, T2DM, triglycerides (TG), HbA1c, and creatinine (Table 4). For the continuous CAP score, significant predictors included age (B = 1.03, SE = 0.34, p = 0.003) and ALT (B = 0.93, SE = 0.27, p = 0.001), indicating that each year of age and each unit increase in ALT raises CAP by 1.03 and 0.93 units, respectively. Platelet count showed a borderline association (B = 0.09, SE = 0.05, p = 0.091). Regarding the continuous LSM score, no predictors reached statistical significance (all p > 0.05); however, sex (male) (B = 2.17, SE = 1.25, p = 0.085) and platelet count (B = 0.01, SE = 0.01, p = 0.089) were observed as borderline, suggesting potential influences that require further exploration. Among categorical outcomes, CAP S3 (advanced steatosis) was significantly associated with WC (OR = 1.09, 95% CI = 1.01-1.18, p = 0.036) and ALT (OR = 1.05, 95% CI = 1.01-1.09, p = 0.013), indicating a 9% and 5% increase in odds per cm and unit increase, respectively. For LSM F3 (advanced fibrosis), significant predictors included age (OR = 1.16, 95% CI = 1.02-1.31, p = 0.021) and HbA1c (OR = 0.12, 95% CI = 0.02-0.67, p = 0.015), with SBP exhibiting a borderline effect (OR = 0.92, 95% CI = 0.84-1.01, p = 0.088). LSM F4 (cirrhosis) was significantly predicted by T2DM (OR = 33.34, 95% CI = 2.35-473.25, p = 0.010), with age (OR = 0.92, 95% CI = 0.84-1.01, p = 0.094) and HbA1c (OR = 2.32, 95% CI = 0.91-5.88, p = 0.077) considered borderline predictors. Notably, extreme OR for sex in the LSM F3/F4 model (e.g., >10⁴⁵, p = 0.998) was excluded due to model instability, indicating potential collinearity or sparse data issues.

**Table 4:** Multivariate regression predictors of hepatic steatosis and fibrosis in physicians attending IDEACON (n = 162). Multivariate regression results, adjusted for all listed variables, are presented for continuous CAP score and LSM score (unstandardised regression coefficients (B), standard errors (SE), p-values) and categorical CAP S3 and LSM F3/F4 (odds ratios (OR), 95% confidence intervals (CIs), p-values) among 162 physicians attending IDEACON. Dashes (—) indicate variables not included in the specific model; sex was excluded from LSM F/F4 models due to model instability OR (>10⁴⁵, p = 0.998). CAP = controlled attenuation parameter; LSM = liver stiffness measurement; WC = waist circumference; BMI = body mass index; SBP = systolic blood pressure; DBP = diastolic blood pressure; ALT = alanine aminotransferase; AST = aspartate aminotransferase; T2DM = type 2 diabetes mellitus; TG = triglycerides; HbA1c = glycated haemoglobin; IDEACON = Integrated Diabetes and Endocrine Academy Conference.

Variable	CAP score	LSM score	CAP S3	LSM F3	LSM F4
	B (SE, p-value)	B (SE, p-value)	OR (95% CI, p-value)	OR (95% CI, p-value)	OR (95% CI, p-value)
Age (years)	1.03 (0.34, 0.003)	-0.01 (0.03, 0.685)	1.02 (0.97-1.07, 0.894)	1.16 (1.02-1.31, 0.021)	0.92 (0.84-1.01, 0.094)
Sex (male)	-6.91 (12.88, 0.592)	2.17 (1.25, 0.085)	1.14 (0.27-4.81, 0.856)	—	—
Height (cm)	2.30 (3.01, 0.447)	0.02 (0.29, 0.934)	0.97 (0.69-1.36, 0.848)	0.44 (0.17-1.16, 0.096)	2.52 (0.68-9.32, 0.167)
Weight (kg)	-1.57 (3.37, 0.642)	-0.03 (0.33, 0.917)	1.00 (0.69-1.46, 0.987)	2.39 (0.84-6.76, 0.101)	0.40 (0.11-1.49, 0.17)
WC (cm)	0.66 (0.49, 0.178)	-0.03 (0.05, 0.468)	1.09 (1.01-1.18, 0.036)	0.93 (0.85-1.03, 0.153)	0.96 (0.88-1.05, 0.374)
BMI (kg/m²)	5.76 (9.34, 0.539)	0.42 (0.91, 0.641)	0.92 (0.32-2.64, 0.875)	0.12 (0.01-1.91, 0.133)	20.89 (1.50-881.25, 0.111)
SBP (mmHg)	-0.03 (0.33, 0.936)	0.04 (0.03, 0.267)	0.99 (0.95-1.03), 0.687	0.92 (0.84-1.01, 0.088)	0.98 (0.91-1.05, 0.488)
DBP (mmHg)	0.47 (0.59, 0.423)	-0.01 (0.06, 0.819)	1.04 (0.97-1.11, 0.311)	1.14 (0.91-1.43, 0.260)	1.02 (0.88-1.17, 0.797)
ALT (U/L)	0.93 (0.27, 0.001)	0.02 (0.03, 0.496)	1.05 (1.01-1.09, 0.013)	1.04 (0.98-1.10, 0.232)	0.96 (0.91-1.02, 0.189)
AST (U/L)	-0.47 (0.50, 0.353)	0.01 (0.05, 0.843)	0.96 (0.90-1.03, 0.242)	1.02 (0.92-1.12, 0.757)	1.06 (0.96-1.17, 0.23)
Platelet count (×10⁹/L)	0.09 (0.05, 0.091)	0.01 (0.01, 0.089)	1.00 (0.99-1.01, 0.558)	0.99 (0.97-1.02, 0.57)	1.01 (1.00-1.02, 0.05)
T2DM (yes)	10.54 (9.58, 0.273)	1.47 (0.93, 0.116)	1.92 (0.68-5.53, 0.221)	3.21 (0.32-32.34, 0.322)	33.34 (2.35-473.25, 0.01)
TG (mg/dl)	0.06 (0.04, 0.162)	-0.00 (0.00, 0.853)	1.00 (1.00-1.01, 0.122)	1.01 (1.00-1.02, 0.155)	1.00 (0.99-1.01, 0.872)
HbA1c (%)	-4.09 (4.87, 0.402)	0.47 (0.47, 0.327)	0.75 (0.42-1.33, 0.327)	0.12 (0.02-0.67, 0.015)	2.32 (0.91-5.88, 0.077)
Creatinine (mg/dl)	-4.80 (10.59, 0.651)	-0.78 (1.03, 0.451)	1.21 (0.41-3.54, 0.727)	0.52 (0.01-27.24, 0.748)	1.22 (0.15-9.64, 0.853)

Additional findings

Among participants with BMI ≥23 kg/m² (n = 143), the prevalence of CAP S3 was 25.4% (36/142) compared with 16.7% (3/18) among those with BMI <23 kg/m²; however, this difference was not statistically significant (Fisher's exact test, p = 0.569). Similarly, the prevalence of LSM F3/F4 was 18.3% (26/142) in participants with BMI ≥23 kg/m² and 5.6% (1/18) in those with BMI <23 kg/m², but this difference also did not reach statistical significance (Fisher's exact test, p = 0.203). WC exhibited a stronger univariate correlation with CAP (r = 0.31) than with LSM (r = 0.25), indicating its potential usefulness in screening for steatosis.

## Discussion

What we know about the subject

MASLD is increasingly recognised as a cardio-metabolic comorbidity, with obesity and visceral adiposity as key drivers [1]. The FIB-4 score, developed initially to detect advanced fibrosis in hepatitis C, has been widely adopted for MASLD due to its simplicity and cost-effectiveness [4]. Its utility was then validated subsequently for patients with MASLD. The American Association for the Study of Liver Disease (AASLD) now endorses FIB-4 for the initial evaluation of fibrosis in patients with MASLD [2].

Studies report high specificity (up to 96%) but variable sensitivity (26-45%) for F3/F4 in MASLD populations, with AUCs typically ranging from 0.76 to 0.85, suggesting moderate discriminatory power [5,11]. However, its performance in detecting steatosis or early fibrosis remains poorly established, with evidence indicating limited sensitivity for CAP-defined steatosis [12]. FibroScan has emerged as a superior non-invasive tool, with CAP and LSM offering direct measures of fat and fibrosis, respectively, and higher accuracy (AUCs >0.85) for advanced stages [7,9]. Yet, its use is constrained by equipment availability and cost, particularly in resource-limited settings [13]. Physicians, despite their medical knowledge, are not immune to MASLD risk factors, with studies showing elevated BMI and metabolic syndrome prevalence in this group [10].

Both the EASL, European Association for the Study of Diabetes (EASD), and European Association for the Study of Obesity (EASO) clinical practice guidelines (2016) and the AASLD practice guidance on nonalcoholic fatty liver disease (NAFLD) (2018, updated 2023) identify 8.0 kPa as a clinically relevant threshold for detecting significant fibrosis (stage F2 or greater) based on LSM by transient elastography [2,14,15].

According to the EASL-EASD-EASO guidelines, an LSM value below 7.0 kPa effectively rules out significant fibrosis, while values between 7.0 and 8.0 kPa fall into an indeterminate zone requiring further evaluation. An LSM exceeding 8.0 kPa suggests the presence of significant fibrosis (F ≥2); values above 9.6 kPa indicate advanced fibrosis (F ≥3), and measurements greater than 13.0 kPa are consistent with cirrhosis (F4) [14].

Similarly, the AASLD practice guidance on NAFLD recommends using an LSM of 8.0 kPa or higher as suggestive of F ≥2. LSM values in the range of 9.6 to 10.5 kPa point toward F ≥3, while values between 12.5 and 14.0 kPa or more are considered indicative of F4 [2,15].

What this study added

This study provides novel insights into FIB-4’s screening utility among 162 physicians attending IDEACON, a unique cohort of healthcare professionals with metabolic risk factors. We found FIB-4 to be largely ineffective for detecting advanced steatosis (CAP S3) and fibrosis (LSM F3/F4), with dismal sensitivities (6.3% for F3/F4, 5.1% for S3) and AUCs near random (0.51 and 0.49), far below prior reports [5,11]. This suggests that FIB-4’s reliance on ALT, AST, and platelet count fails to capture early fibrosis in this population, even with its high specificity (95.7%). The poor performance of FIB-4 observed in this study should also be interpreted within the context of the study population. Participants were physicians attending a conference focused on diabetes and endocrinology, many of whom had obesity and other metabolic risk factors. Consequently, the findings may reflect the characteristics of this selected cohort rather than a universal limitation of FIB-4 across all MASLD populations. Further studies in community-based and more diverse populations are required to determine the generalizability of these observations. Multivariate analyses identified WC and ALT as significant predictors of CAP S3 (ORs = 1.09 and 1.05, p < 0.05) and age and HbA1c for LSM F3 (ORs = 1.16 and 0.12; p < 0.05), with T2DM strongly linked to LSM F4 (OR = 33.34, p = 0.010). The elevated prevalence of CAP S3 (25.69%) and LSM F3/F4 (18.06%) in obese participants (BMI ≥23 kg/m²) underscores the need for dual CAP & LSM screening in at-risk subgroups, as FIB-4 alone missed most cases. These findings highlight WC’s screening potential and physicians’ own MASLD burden, adding to the limited literature on this population [10]. The present findings should be interpreted in the context of transient elastography-based fibrosis assessment, as histological confirmation was not available.

Practical implications

The findings of this study have important implications for MASLD screening strategies. Although combined CAP and LSM assessment identified a substantial burden of steatosis and fibrosis that was not detected by FIB-4 alone, universal implementation of transient elastography may not be feasible in many healthcare settings because of equipment costs, operator requirements, and limited availability. Therefore, our findings should not be interpreted as supporting population-wide FibroScan screening. Rather, they suggest that targeted CAP and LSM assessment may be most beneficial in individuals with established metabolic risk factors such as obesity, increased WC, and T2DM.

In resource-limited settings, a pragmatic approach may involve initial clinical risk assessment using anthropometric measures and metabolic risk profiling, followed by selective referral for transient elastography in higher-risk individuals. Because WC emerged as an independent predictor of advanced steatosis in our study, this simple and inexpensive measure may help identify individuals who could derive the greatest benefit from further hepatic evaluation. Future cost-effectiveness studies are needed to determine the optimal integration of transient elastography into MASLD screening pathways.

Study limitations

We acknowledge that this cross-sectional study was carried out using convenience sampling and thus bears the baggage of important methodological limitations such as selection bias. The sampled individuals were all physicians and thus may have systematically differed from the broader population in key demographic, clinical, and behavioural characteristics. This overrepresentation of a specific subgroup may have led to distortion of observed associations and outcomes. The outcome of this study thus suffers from limited generalisability. No formal sample size estimation was performed prior to study initiation because the study was designed as an opportunistic, conference-based screening programme. Consequently, certain subgroup analyses, particularly those involving advanced fibrosis outcomes, may have been underpowered.

Other limitations warrant consideration. Because participation was voluntary, physicians who were more health-conscious or concerned about metabolic health may have been more likely to participate, introducing the possibility of self-selection bias. Individuals with known metabolic risk factors, greater health awareness, or concerns regarding obesity, diabetes, or liver health may have been more likely to participate than their counterparts. Such self-selection could have inflated the observed prevalence of steatosis and fibrosis relative to the general physician population. Conversely, physicians attending a professional conference may also represent a health-conscious subgroup with better access to healthcare and preventive services. Therefore, the prevalence estimates reported in this study should not be interpreted as representative of either all physicians or the broader MASLD population. All statistical outputs and dataset values were independently re-audited against the cleaned source dataset prior to resubmission to address prior reporting inconsistencies. The cross-sectional design precludes causality inferences between predictors (e.g., WC and T2DM) and hepatic outcomes. The convenience sample of physicians attending IDEACON, predominantly male (86.34%), may not generalise to broader populations and potentially skew sex-specific findings (e.g., model instability in LSM F3/F4). The multivariable logistic regression analyses should be interpreted with caution because the number of fibrosis events, particularly for LSM F4, was relatively small compared with the number of covariates included in the models. This may have resulted in a low events-per-variable ratio, increasing the risk of model overfitting, coefficient instability, and imprecise effect estimates. The wide confidence intervals and occasional extreme odds ratios observed in some models likely reflect these limitations. Consequently, the multivariable findings should be regarded as exploratory and hypothesis-generating and require validation in larger cohorts. Third, liver biopsy was not performed, and therefore histological confirmation of steatosis and fibrosis was unavailable. Although transient elastography (CAP and LSM) is a validated and widely accepted non-invasive modality for assessing hepatic steatosis and fibrosis, it remains an indirect surrogate for histological disease severity. Consequently, the diagnostic performance measures reported in this study, including sensitivity, specificity, predictive values, and AUCs, reflect the ability of FIB-4 to identify transient elastography-defined hepatic abnormalities rather than biopsy-confirmed fibrosis. This should be considered when interpreting the findings [7]. However, FibroScan has been the defining diagnostic parameter in many studies and remains a validated tool. Additionally, a minimum fasting period of three hours was uniformly applied before FibroScan assessment; some studies have recommended longer fasting durations prior to transient elastography. Therefore, a modest influence of postprandial physiological variation on CAP and LSM measurements cannot be completely excluded. Fourth, significant kit-to-kit and reagent-based variations exist in liver enzyme estimation, leading to differences in results between various liver function tests. Variations in AST and ALT results from different kits could lead to different FIB-4 scores. Finally, small subgroup sizes (e.g., LSM F4, n = 15) and extreme ORs suggest potential overfitting or sparse-data bias, warranting cautious interpretation.

Study strengths

This study’s strengths include its focus on a novel cohort of physicians offering a unique lens on MASLD risk in healthcare professionals. Because IDEACON primarily focuses on diabetes and metabolic diseases, this cohort likely represented a population with heightened metabolic risk awareness, making the observed burden of MASLD particularly noteworthy. Transient elastography provided precise CAP and LSM measurements, enhancing outcome reliability over serum-based scores alone [9]. Multivariable analyses were performed to explore independent associations between clinical variables and hepatic outcomes after adjustment for potential confounders. The rigorous statistical approach, including ROC analysis and correlation coefficients, strengthens the evidence for FIB-4’s limitations and dual screening’s necessity in at-risk groups.

## Conclusions

In conclusion, this study demonstrates that the FIB-4 score is an inadequate screening tool for detecting advanced fibrosis in physicians with metabolic risk factors, exhibiting poor sensitivity and discriminatory power. Dual screening with CAP and LSM is essential for identifying at-risk individuals, particularly those with obesity or increased WC, where WC and T2DM emerged as key predictors. These findings advocate for targeted liver health assessments in high-risk populations, including healthcare professionals, to improve early detection and management of MASLD. Future longitudinal studies with more extensive, diverse cohorts and histological validation are needed to confirm these results and refine screening strategies.

## References

[1] Younossi ZM, Koenig AB, Abdelatif D, Fazel Y, Henry L, Wymer M (2016). Global epidemiology of nonalcoholic fatty liver disease—meta-analytic assessment of prevalence, incidence, and outcomes. Hepatology.

[2] Chalasani N, Younossi Z, Lavine JE (2018). The diagnosis and management of nonalcoholic fatty liver disease: practice guidance from the American Association for the Study of Liver Diseases. Hepatology.

[3] Castera L, Friedrich-Rust M, Loomba R (2019). Noninvasive assessment of liver disease in patients with nonalcoholic fatty liver disease. Gastroenterology.

[4] Sterling RK, Lissen E, Clumeck N (2006). Development of a simple noninvasive index to predict significant fibrosis in patients with HIV/HCV coinfection. Hepatology.

[5] McPherson S, Stewart SF, Henderson E, Burt AD, Day CP (2010). Simple non-invasive fibrosis scoring systems can reliably exclude advanced fibrosis in patients with non-alcoholic fatty liver disease. Gut.

[6] Chen M, Guo C, Ouyang K, Liu N (2024). Diagnostic role of the Fibrosis-4 index and nonalcoholic fatty liver disease fibrosis score as a noninvasive tool for liver fibrosis scoring. Medicine (Baltimore).

[7] Wong VW, Vergniol J, Wong GL (2010). Diagnosis of fibrosis and cirrhosis using liver stiffness measurement in nonalcoholic fatty liver disease. Hepatology.

[8] European Association for the Study of the Liver (2021). EASL clinical practice guidelines on non-invasive tests for evaluation of liver disease severity and prognosis - 2021 update. J Hepatol.

[9] Eddowes PJ, Sasso M, Allison M (2019). Accuracy of FibroScan controlled attenuation parameter and liver stiffness measurement in assessing steatosis and fibrosis in patients with nonalcoholic fatty liver disease. Gastroenterology.

[10] Umasankari S, Aishwarya S, Aishwarya SK, Bhardwaj S, Pavithra RB, Ray S, Vinodhini VM (2024). Exploring the epidemiology and awareness of metabolic dysfunction-associated steatotic liver disease (MASLD) among health sciences students in an academic health care institute in India. Metabol Open.

[11] Albert SG, Wood EM (2024). FIB-4 as a screening and disease monitoring method in pre-fibrotic stages of metabolic dysfunction-associated fatty liver disease (MASLD). J Diabetes Complications.

[12] Chang M, Chang D, Kodali S, Harrison SA, Ghobrial M, Alkhouri N, Noureddin M (2024). Degree of discordance between FIB-4 and transient elastography: an application of current guidelines on general population cohort. Clin Gastroenterol Hepatol.

[13] Younossi ZM, Paik JM, Henry L, Stepanova M, Nader F (2025). Pharmaco-economic assessment of screening strategies for high-risk MASLD in primary care. Liver Int.

[14] European Association for the Study of the Liver (EASL), European Association for the Study of Diabetes (EASD), European Association for the Study of Obesity (EASO) (2016). EASL-EASD-EASO clinical practice guidelines for the management of non-alcoholic fatty liver disease. J Hepatol.

[15] Rinella ME, Neuschwander-Tetri BA, Siddiqui MS (2023). AASLD practice guidance on the clinical assessment and management of nonalcoholic fatty liver disease. Hepatology.

